# Extract of a Letter to the Baltimore Editor, from Mr. Andrew Wilson, of Edinburgh

**Published:** 1849-07

**Authors:** 


### ARTICLE III.

*Extract of a letter to the Baltimore Editor, from* Mr. Andrew Wilson of Edinburgh, dated May 16, 1849.

Sir:—Having lately been perusing your valuable work on dental surgery, I find that the method recommended for backing mineral teeth, differs considerably from that practiced by me, and which as I think preferable, I will briefly describe, so that you may judge for yourself. After having partially fitted the tooth to the plate, take a piece of thick platina foil, (as thick as can be used conveniently,) and pressing it against the back of the tooth, perforate it where it is marked by the pins, then cut it into the shape of the back as wished to be, and press it as closely as possible to the back of the tooth.

It will now be requisite to apply a little borax to the platina pins which come through the back, and placing the tooth with its face downwards upon a thin piece of pumice, covered with dry plaster of paris, put several pieces of gold (according to the thickness required) upon the platina back, slowly heat it, gradually raising the heat till it is considered safe to melt the gold with the blow-pipe, when upon continuing the blast the gold will rapidly flow over the whole platina surface, incorporating so accurately with the pins in the tooth, that I have never seen a case of their being withdrawn when the tooth has been broken during the whole time it has been in use here, (nearly eight years,) they always remaining firmly fixed in the backing upon the plate.

After the backing has been run and the tooth allowed to cool slowly, it is filed to the requisite thickness and shape, when being closely fitted to the base it is finally soldered to the plate, as described in your work. We generally use a mixture of fine sand (white) and plaster of paris, equal parts, for encasing the piece and also place a thin curved strip of platina in front of the teeth, having a layer of the above mixture on both sides of it, so that should the plaster crack in the soldering, (although it is less liable to do so than plaster alone,) the platina keeps the teeth from shifting their places. The whole time occupied in heating and backing a tooth occupies about half an hour, and when several are doing at once a very little longer.

We generally employ the same method in making ready the base of a mineral pivot, instead of the tedious process of making casts and striking a plate, and fitting a great deal better to the root, (in consequence of the contraction of the metal during its cooling,) of course all that is required for attaching the pivot, is to perforate the platina base and passing the gold wire through it, solder it to the platina and run gold over the surface of the platina at the same time.

The following are the formula computed and employed by my father, (the late Mr. William Wilson, dentist,) for either reducing or raising the fineness of gold to any required standard.

1st, Let $a$ represent twenty-four carat of fine gold.
$w$ its weight, and $b$ the required carat, then the weight of alloy to be added is $\frac{a-b}{b} \times w$

2d, Let $a$ represent twenty-four carat gold.
$b$ the required carat.
$c$ that to be raised, and
$w$ its weight. Then will $\frac{b-c}{a-b} \times w$ be the weight of fine gold required to be added. Thus,

1 oz. 16 carat=13$\frac{1}{3}$ g. and 6$\frac{2}{3}$ *a* (2.1) and adding 1 oz. =20 g. We have got 33$\frac{1}{3}$ g. and 6$\frac{2}{3}$ *a* (5.1) or 2 oz. 20 carat as by the formula $\frac{20-16}{24-20} \times w = 1 \times w$ or an equal weight of fine gold.

Of course the proportions of the alloying metals depend upon use, color, and hardness required.

The above methods may be in more general use, that I know of, as in *this country*, from absurd and mistaken notions. No one can say what methods another practitioner may employ.

